# An Intraoperative Encounter With Langer’s Axillary Arch and Its Clinical Significance

**DOI:** 10.7759/cureus.83601

**Published:** 2025-05-06

**Authors:** Elizabeth Tan, Michael Issac, Peter Barry

**Affiliations:** 1 General and Breast Surgery, The Northern Hospital, Melbourne, AUS; 2 Breast Surgery, The Northern Hospital, Melbourne, AUS

**Keywords:** axilla anatomy, axillary surgery, langer's arch, langer's axillary arch, sentinel node biopsy

## Abstract

Langer’s axillary arch (LAA) is the most common accessory muscle encountered during axillary surgery. We present a 55-year-old lady who underwent a right wide local excision and sentinel lymph node biopsy. Intraoperatively, we identified an aberrant muscle slip traversing the axilla, anterior to the neurovascular structures. It originated from the latissimus dorsi muscle and inserted into the pectoralis major. This report explores the current knowledge of LAA and discusses the importance of recognising it intraoperatively in the era of minimisation of axillary surgery. Implications of failing to recognise it include suboptimal regional control, compromised oncological treatment, or neurovascular injuries.

## Introduction

Despite the improved survival of breast cancer patients, the incidence of breast cancer, and therefore axillary surgery, is rising [1]. Knowledge regarding anatomical variations in oncological lymphadenectomy for breast cancer and melanoma is crucial for safe surgical practice. It is also applicable in breast reconstruction and axillary bypass procedures [2].

Langer’s axillary arch (LAA), a remnant of the panniculus carnosus, is the most well-known variation of axillary anatomy [3]. It is typically a muscular or fibromuscular slip originating from the latissimus dorsi (LD) muscle, and crosses through the axilla anterior to neurovascular structures with varied insertion [2-4].

## Case presentation

A 55-year-old lady was diagnosed with a right Breast Imaging Reporting and Data System (BIRADS) 4, 14×11 mm spiculated retro-areolar mass. Core biopsy confirmed a grade 2 invasive ductal carcinoma (IDC); estrogen and progesterone receptor positive, HER2 negative, and Ki67 5%.

**Figure 1:**
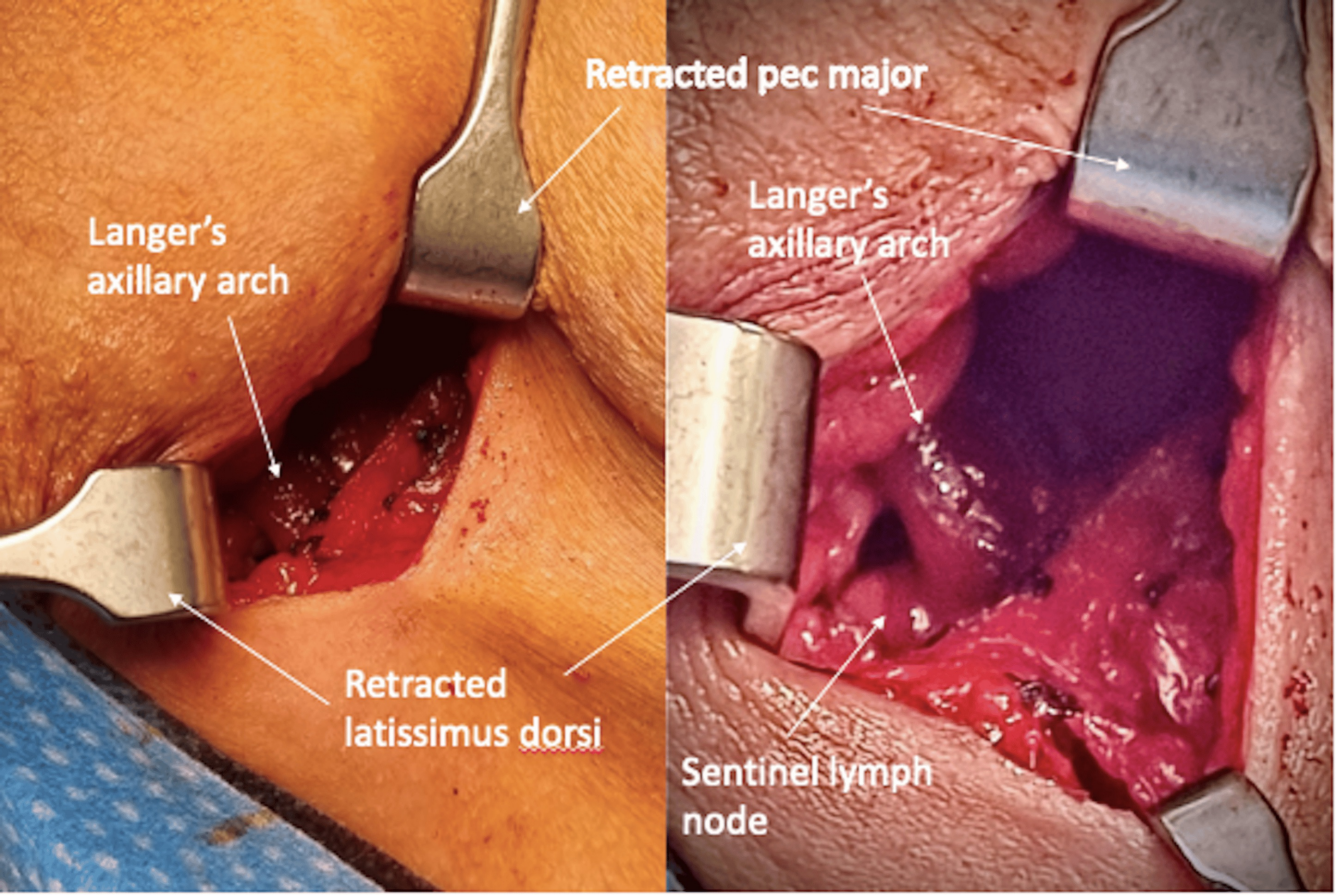
The left image depicts the incidental finding of Langer’s axillary arch exposed after clavipectoral fascia incision. The right image highlights the lymph node situated posterior to the axillary arch.

She underwent right wide local excision and sentinel lymph node biopsy (SLNB). Preoperative lymphoscintigram mapped to the ipsilateral axilla. A 4 cm incision was made just below the hair-bearing crease in the axilla following gamma probe localisation. The clavipectoral fascia was incised. An aberrant band of muscle was identified, crossing the axilla from the LD and inserting into the posterior surface of the pectoralis major. This muscle variant was confirmed as LAA, with axillary neurovascular structures and lymph nodes posteromedial to it (Figure 1). There was no compression of adjacent structures.

The LAA was divided with diathermy, allowing adequate access to the sentinel lymph node medial to and beneath the arch. LAA retraction alone would still have limited the field of dissection and compromised the yield of sentinel lymph nodes. No other variations were identified, and the patient had an uneventful recovery. Final histopathology confirmed an 18 mm IDC with one sentinel lymph node clear.

## Discussion

Current literature reports three accessory muscles identified in axillary surgery, LAA, pectoralis quartus, and chondroepitrochlearis muscles [5]. These accessory muscle variations all traverse the axilla, with LAA being the most common [5]. The pectoralis quartus usually originates from the fifth or sixth rib and inserts into the bicipital groove of the humerus, whilst the chondroepitrochlearis muscle arises from the pectoralis major and inserts into the epicondyle of the humerus [5]. LAA was first described by Ramsay in 1795, then eponymously named after Karl Langer, who further detailed its anatomy in 1846 [2-5]. It is often identified intraoperatively during axillary surgery for the treatment of breast cancer and upper limb/trunk melanomas [5-7]. It has a reported incidence of 7% [6-7].

Usually unilateral, LAA has a constant origin from the anterior border of the LD and crosses over the axillary neurovascular bundle [6-7]. There is wide variation in size and insertion, the most common being the inferior edge of the pectoralis major [3]. This insertion is defined by Testut in 1884 as ‘complete’ [3]. ‘Incomplete’ insertions include the axillary fascia, coracoid process, coracobrachialis, or biceps brachii [3]. LAA is supplied by a branch of the lateral thoracic artery and is innervated by the lateral pectoral nerve, medial pectoral nerve, thoracodorsal nerve, or intercostobrachial nerve with varying frequencies [6-7]. It is classified into two groups, type 1 (muscular form) and type 2 (fibrous form) [6-7]. Our patient had a unilateral type 1 LAA.

Preoperative detection is limited in asymptomatic patients [8-9]. LAA can present as a palpable axillary mass or be mistaken for an enlarged lymph node [8-9]. It can also manifest with symptoms of paraesthesia, pain, and a swollen upper limb when neurovascular entrapment occurs, as seen in hyperabduction syndrome and brachial plexus impingement [8-9]. It can sometimes be identified on axillary ultrasound as an isoechoic muscular structure, or as an ovoid density or band-like structure on a mediolateral oblique mammogram [9-11]. MRI is the ideal imaging modality to assess the soft tissue structure of LAA and its anatomic relations [12,13].

LAA is additionally clinically significant as it can be associated with further anomalies such as two axillary veins, brachial plexus variations, and absence of the intercostobrachial nerve [10]. Secondly, the presence of an LAA can obscure axillary nodes laterally and/or posteriorly [3-6,11]. This was evident in our patient, with the SLN located posterior to the LAA. Failure to identify and remove these axillary nodes during SLNB, TAD, or axillary node dissection could increase the risk of regional relapse in breast cancer and melanoma, as well as cause false down-staging that impacts decision-making for adjuvant systemic therapy [3-6,11]. In our case of a postmenopausal woman with a small and low-grade luminal A cancer, omission of SLNB in the presence of LAA variation was considered. However, when comparing the benefit of further treatment options in the event of a positive SLNB versus the minimal morbidity of LAA division (such as decreased range of movement and increased muscle bleeding), a decision to proceed with SLNB was made.

Thirdly, LAA can be mistaken for the LD, leading to dissection in the supra-axillary plane. This dissection can result in axillary artery and brachial plexus cord injury [11]. Fourthly, on arm hyperabduction, the stretched LAA obscures and shifts the sentinel lymph nodes higher [11]. This was evident in our dissection, with difficult access to the sentinel lymph nodes that were now in a higher position; hence, we divided the LAA, leading to successful SLNB.

There is debate in the literature regarding routine division of the LAA [9,11,14]. No morbidity has been identified with LAA division to facilitate dissection during sentinel node biopsy, axillary dissection, vascular bypass surgery, and breast reconstruction with a LD myocutaneous flap [14]. It also improves visualisation of axillary structures and anatomical landmarks.

Another reason for LAA transection is the potential for postoperative swelling, leading to upper limb lymphedema or axillary vein thrombosis [14]. Weninger JT et al.'s large cadaveric cohort identified that 64% had the potential to touch or compress the neuromuscular axillary bundle upon arm abduction and external rotation [15].

Das S et al.'s novel classification of LAA, based on insertion and neurovascular compression (Type 1-3), can be used to guide LAA transection or preservation during axillary dissection [16]. LAA transection is recommended in Type 1 (pectoralis major insertion) and if compression is evident (Types 1C, 2C, and 3C) [16]. Taterra D et al.'s meta-analysis defined compression as less than 2 cm clearance between the arch and axillary vessels with one finger or instrument [14].

Baskin AS et al. reported successful exposure of the axilla in their SLNB for a melanoma patient without LAA transection [17]. The LAA encountered was a large, fan-like shape [17]. They describe a muscle-splitting technique and retraction that enabled successful access for dissection [17]. Hence, with the benefit of LAA transection outweighing the risk of preservation, we recommend transection to aid axillary exposure and ensure safe axillary surgery.

Given the current worldwide trend to de-escalate the extent of axillary surgery from full dissection to SLNB or TAD [18], operative exposure is necessarily more limited; therefore, careful and early identification of aberrant anatomy in a more restricted operative field is essential. Early identification is therefore of paramount importance to ensure effective and safe surgery with identification and removal of the selected lymph node sample.

## Conclusions

This case highlights the diagnostic challenge of LAA. Although LAA is relatively rare, it is essential that surgeons are aware of its anatomy, associated variations, and operative implications. We recommend division of the LAA to achieve optimal axillary dissection. This avoids intraoperative confusion and injury to the axillary vein and/or long thoracic and thoracodorsal neurovascular structures. Inadequate superficial axillary lymph node dissection or SLNB can also lead to inaccurate oncological management of breast cancer patients.
